# Comprehensive Analysis of Human microRNA–mRNA Interactome

**DOI:** 10.3389/fgene.2019.00933

**Published:** 2019-10-08

**Authors:** Olga Plotnikova, Ancha Baranova, Mikhail Skoblov

**Affiliations:** ^1^Laboratory of Functional Genome Analysis, Moscow Institute of Physics and Technology, Moscow, Russia; ^2^Laboratory of Functional Genomics, Research Centre for Medical Genetics, Moscow, Russia; ^3^School of Systems Biology, George Mason University, Fairfax, VA, United States

**Keywords:** microRNA, regulation of gene expression, microRNA–mRNA interactions, microRNA-binding sites, miRNA-target RNA duplexes, web tool for searching microRNA-binding regions

## Abstract

MicroRNAs play a key role in the regulation of gene expression. A majority of microRNA–mRNA interactions remain unidentified. Despite extensive research, our ability to predict human microRNA-mRNA interactions using computational algorithms remains limited by a complexity of the models for non-canonical interactions, and an abundance of false-positive results. Here, we present the landscape of human microRNA–mRNA interactions derived from comprehensive analysis of HEK293 and Huh7.5 datasets, along with publicly available microRNA and mRNA expression data. We show that, while only 1–2% of human genes were the most regulated by microRNAs, few cell line–specific RNAs, including EEF1A1 and HSPA1B in HEK293 and AFP, APOB, and MALAT1 genes in Huh7.5, display substantial “sponge-like” properties. We revealed a group of microRNAs that are expressed at a very high level, while interacting with only a few mRNAs, which, indeed, serve as their specific expression regulators. In order to establish reliable microRNA-binding regions, we collected and systematically analyzed the data from 79 CLIP datasets of microRNA-binding sites. We report 46,805 experimentally confirmed mRNA–miRNA duplex regions. Resulting dataset is available at http://score.generesearch.ru/services/mirna/. Our study provides initial insight into the complexity of human microRNA–mRNA interactions.

## Introduction

MicroRNAs are small noncoding RNAs that associate with Argonaute (*AGO*) protein to form a silencing complex, which then regulates a gene expression (Jonas and Izaurralde, 2015). MicroRNAs accomplish essential post-transcriptional regulatory step of gene expression regulation through either the degradation of a transcript or the inhibition of translation and are involved in key cellular processes, such as apoptosis, proliferation, or differentiation (He and Hannon, 2004). Hence, dysregulation of microRNAs may result in the development of a disease or in a malignant transformation (Weiss and Ito, 2017). According to some estimates, nearly all mature sequences of coding transcripts contain potential sites for microRNA regulation (Bartel, 2004; Friedman et al., 2009).

Human genome encodes approximately 2,600 mature microRNAs (miRBase v.22) and, according to GENCODE data (v.29), more than 200,000 of transcripts, including isoforms with slight variations. A particular microRNA may target many different mRNAs (Selbach et al., 2008); a particular messenger RNA may bind to a variety of microRNAs, either simultaneously or in context-dependent fashion (Uhlmann et al., 2012). Notably, the target regions for particular microRNAs commonly cluster together, thus resulting in the cooperative repression effect (Grimson et al., 2007; Sætrom et al., 2007). The mapping of microRNA–mRNA interactions is far from being complete due to the recognized challenge of computational prediction of mRNA–microRNA interactions.

In our previous study, we showed that the outputs generated by commonly used microRNA–mRNA interactions predicting software differ substantially, while failing to pinpoint experimentally confirmed microRNA-binding regions correctly (Plotnikova and Skoblov, 2018). Nowadays, many tools for the prediction microRNA–mRNA interactions are in development, all with different underlying algorithms (Agarwal et al., 2015; Gumienny and Zavolan, 2015; Lu and Leslie, 2016; Riffo-Campos et al., 2016). Among most advanced algorithms, we should highlight the ones taking into account expression levels of both the microRNAs and their targets. Notably, the changes in expression of microRNA may also affect expression levels of other, non-target mRNAs—for example, due miRNA targeting of their upstream regulators. Consequently, newer, more comprehensive approaches—for example, MiRImpact (Artcibasova et al., 2016), PanMiRa (Li and Zhang, 2014), and ProMISe (Li et al., 2014), aim at explaining complex phenotypes by performing analysis of each microRNA along with its direct and indirect targets.

Experimental identification of direct microRNA targets remains a crucial step in attaining reliable prediction results. There are two main groups of the experimental approaches for a direct identification of microRNA–mRNA interactions. The first approach relies on a construction of reporter gene assays and one-by-one evaluation of possible interactions between the microRNA and its cognate mRNA region of interest through measuring the activity of the reporter (Steinkraus et al., 2016). Another group of techniques comprises involves a coupling of a cross-linking with immunoprecipitation (CLIP); this group represented by variety of the protocols including PAR-CLIP, iCLIP, HITS-CLIP, and others (Licatalosi et al., 2008; Steinkraus et al., 2016). CLIP group of methods identifies the microRNA-binding regions in target mRNAs only, while information about pairing of a particular microRNA with a particular mRNA region remains obscure.

Two modifications of AGO-CLIP based technology were developed specifically for identifying microRNAs ligated to their endogenous mRNA targets as part of chimeric molecules. To date, AGO-CLIP-based evaluations of microRNA–mRNA interactomes were executed only in two human cell lines. Helwak and colleagues applied so-called cross-linking ligation and sequencing of hybrids, or CLASH, to HEK293 cell line, retrieving more than 18,000 high-confidence microRNA–mRNA interactions (Helwak et al., 2013). Later, Moore and colleagues used another variety of AGO-CLIP termed CLEAR (covalent ligation of endogenous Argonaute-bound RNAs)-CLIP for the study of microRNA interactome in Huh7.5 cell (Moore et al., 2015). CLASH and CLEAR-CLIP techniques closely resemble each other, with the only difference that CLASH protocol employs HEK293 cell line over-expressed AGO1, while CLEAR-CLIP targets endogenous AGO allowing experimenting with any cell line. Thus, CLEAR-CLIP does not require full denaturation of AGO and involves a single purification step. It is of note that both publications cited above concentrated on the development of the experimental protocol and subsequent evaluation of the technical aspects of analytic procedure, rather than on extracting biological insights from the data collected.

**Figure 1:**
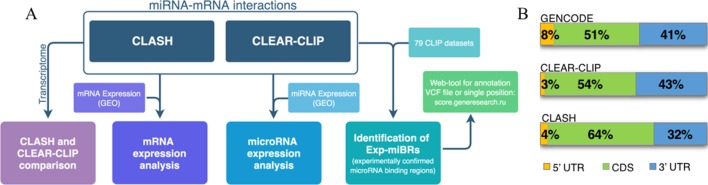
Analysis of human microRNA–mRNA interactome. **(A)** Flowchart describing main steps of human microRNA–mRNA interactome data analysis. **(B)** Distribution of the summarized lengths of 3’UTR, CDS, or 5’UTR mRNA regions in CLEAR-CLIP, CLASH, and GENCODE, respectively.

A flowchart at **Figure 1A** represents the methodology for analysis of microRNA–mRNA human interactome employed in this study. We aggregated various experimental data on human miRNA–mRNA interactions and then investigated how expression levels of each studied microRNA and each of its cognate mRNAs correlate, and whether the behavior of miRNA–mRNA pairs depends on a cell line context. In order to do this, we analyzed together (i) sequences and abundance of microRNA and their target mRNAs in CLASH dataset for HEK293 cell line and in CLEAR-CLIP dataset for Huh7.5 cell line, and (ii) expression level of microRNAs and target mRNAs in HEK293 and in Huh7.5 cell lines. Second, we performed systematic extraction of credible, experimentally confirmed microRNA-binding regions across CLASH/CLEAR-CLIP datasets and in 79 additional CLIP datasets and present them here as a collection.

## Materials and Methods

### microRNA–mRNA Interactions

microRNA–mRNA interactome data were extracted from previously published CLASH (Helwak et al., 2013) and CLEAR-CLIP (Moore et al., 2015) datasets. CLASH data provide transcriptome coordinates for 18,514 miRNA–mRNA interactions, while CLEAR-CLIP dataset include genome coordinates (version hg18) for 32,712 interactions. Using Ensembl API (https://rest.ensembl.org/, Yates et al., 2014), the coordinates of CLASH microRNA–mRNA-interacting regions were transformed into genome coordinates. For 36 interactions, the transforming of their coordinates failed and, in total, we revealed 18,478 microRNA–mRNA interactions in 22,030 genome regions (all interactions were located in mRNA regions, with 19% being divided between two exons and 36 interactions of three exons). We used LiftOver (https://genome.ucsc.edu/cgi-bin/hgLiftOver, Kuhn et al., 2012) to transform CLEAR-CLIP interactome data from hg18 genome version into hg19. A total of two interactions failed to transform. Hence, resultant amount of interactions equaled 32,710. Genomic regions (CDS, 3’UTR, 5’UTR, intronic, intergenic, etc.) were annotated by wAnnovar (Wang et al., 2010; Yang and Wang, 2015).

To compare CLASH and CLEAR-CLIP data, CLEAR-CLIP dataset was reduced to microRNA–mRNA interactions mapped to the expressed transcriptome (n = 10,032). For each of the sets of genomic regions (3’UTR, CDS, and 5’UTR) found in miRNA bound regions present in CLASH and CLEAR-CLIP, their average length (mean) was comparable to that calculated for all protein-coding transcripts (N = 59,900) downloaded from GENCODE, version 24 (Frankish et al., 2018).

To calculate expected overlap between CLASH and CLEAR-CLIP datasets, five independent CLASH-like and CLEAR-CLIP-like datasets were generated. For each simulation, binding regions were randomly selected from CLASH/CLEAR-CLIP transcripts in amounts equal to detected amount of interactions.

CLASH and CLEAR-CLIP datasets were utilized to evaluate the amount of interactions for each of the genes as a sum of all interactions between microRNAs and mRNA encoded by each gene.

### mRNA Expression

Publicly available RNAseq datasets GSE68611 (Murakawa et al., 2015) and GSE64677 (Luna et al., 2015) were used for extracting and examining gene sets expressed in HEK293 and Huh7.5 cell lines. Each of these datasets includes two biological replicates. Initial quality control of sequencing outputs was performed using FastQC (www.bioinformatics.babraham.ac.uk/projects/fastqc). Next, we used kallisto (Bray et al., 2016) to map raw reads to the human reference transcript sequences (GENCODE, 28 version).

First, in each experiment, we calculated the gene expression levels as the sum of expression levels for individual gene transcripts. Second, we took the mean value for each gene between two processed datasets in each of the two cell lines. Finally, we kept only genes that had expression more or equal to 1 tpm as total value and that had expression level of at the level at least 1 tpm in one of the two experiments.

In order to compare only genes reliably expressed both in HEK293 and Huh7.5 cells, only the genes expressed at levels of more than 10tpm or higher were included.

Gene functions were interpreted using PANTHER toolkit Version 12.0 (http://www.pantherdb.org/tools). We used InteractiVenn tool (Heberle et al., 2015) to create Venn diagrams in our analysis.

### microRNA Expression

We downloaded microRNA expression data from the GEO database: two experimental replicates for HEK293 cell line (GSE75136 (Wissink et al., 2016)) and three experimental replicates for Huh7.5 cell line (GSE74014 (Bandiera et al., 2016)). The correlations of experimental results obtained in two cell lines were calculated by the Spearman’s procedure. We used the R package “DeSeq2” (Love et al., 2014) to normalize microRNA expression. Particular microRNA was considered as expressed if its expression levels were of three or more counts.

CLASH and CLEAR-CLIP datasets were used to calculate the amount of interactions for each microRNA. The correlation of the amounts of interactions formed by microRNAs and their expression levels were estimated using the Spearman correlation coefficient.

In order to calculate conservative phyloP scores, for all microRNAs, we downloaded the coordinates of the mature microRNAs from miRBase (Kozomara and Griffiths-Jones, 2014) (release 22, coordinates corresponded to the GRCh38 human reference genome). Next, we used UCSC table browser (Karolchik et al., 2004) to obtain phyloP conservative values across 20 vertebrates for all mature microRNAs. For each group of microRNAs, the mean value between the phyloP scores was calculated.

### CLIP Data

We collected 79 CLIP datasets (**Supplementary Table 1**) from the POSTAR database (Hu et al., 2016). Raw data of these CLIP datasets were initially pre-processed by FASTX-Toolkit (http://hannonlab.cshl.edu/fastx_toolkit) and then were processed by specialized tools for different CLIP-seq technologies: PARalyzer (Corcoran et al., 2011) for PAR-CLIP datasets (N = 18) and CIMS (Moore et al., 2014) for HITS-CLIP datasets (N = 61). We used python to analyze all microRNA-binding regions from CLIP datasets together with microRNA–mRNA interactions from CLASH and CLEAR-CLIP. In total, all regions were merged in six million nucleotides, and each position was characterized by the following parameters: list of supported experiments (GEO GSM ID), their corresponding cell lines and list of interacted microRNAs (if accessible). We used wAnnovar to annotate genes and their parts (CDS, 3’UTR, 5’UTR, intronic, etc.).

### microRNA-Binding Regions

Our analysis of CLIPs, CLASH, and CLEAR-CLIP revealed 156,000 regions. We used a custom python script to select experimentally confirmed microRNA-binding regions (Exp-MiBR). Exp-MiBR was defined as a region that had a subsequence of length L = 10, whereas each nucleotide (position) in this subsequence had been supported by at least n = 2 different datasets or chimeras. We estimated the amount of Exp-MiBRs for all combination of length and amount of supported datasets/chimeras in ranges: L = 1–25 and n = 1–10 (**Supplementary Table 2**).

### Exp-MiBR Application

We characterized each Exp-MiBR (total amount = 46,805) by the following parameters: gene information, amount and list of supported experiments (GEO GSM ID) and their corresponding cell lines, and list of interacted microRNAs (if accessible).

Besides that all the Exp-MiBRs with the corresponded information are available as **Supplementary Table 3**, we also provide an open-access web tool *via*http://score.generesearch.ru/services/mirna. As input, the tool requires any VCF file (v4.0 or 4.1), no more than 20MB or a single (point) genome coordinate. The file or coordinate could be recorded in human genome assembly version 38 or 19.

### Web Tool for Searching Exp-MiBRs

All microRNA-binding regions identified as experimentally confirmed (Exp-MiBR) and reported in this paper (**Supplementary Table 3**) may be searched by a web tool available online: http://score.generesearch.ru/services/mirna/.

## Results

### Comparison of High-Throughput microRNA–mRNA Interactions From CLASH and CLEAR-CLIP Datasets

First, the sets of microRNA–mRNA interactions retrieved in HEK293 and in Huh7.5 by CLASH (Helwak et al., 2013) and CLEAR-CLIP (Moore et al., 2015) protocols were compared, respectively, to hg19 genome references. Although CLASH and CLEAR-CLIP techniques are somewhat similar, CLEAR-CLIP study (N = 32,710) revealed almost two times more interactions than CLASH study (N = 18,478). One of the reasons for this may be due to the differences in the data processing procedures. While CLASH sequences were aligned to the mature transcriptome, CLEAR-CLIP data have been mapped to human genome. Because of that, CLEAR-CLIP technique was capable to highlight additional interaction sites located in the introns and the intergenic regions (∼70% of all interactions).

To enable the comparison, we focused our analysis on miRNA-binding regions residing within the mature transcriptome (**Supplementary Table 4**). Because of that, CLEAR-CLIP dataset was limited to about one-third of its entries (n = 10,032). Further analysis estimated that approximately 2–3% of the total length of all expressed protein-coding transcripts serve as a target for one or another microRNAs in either CLASH or CLEAR-CLIP datasets. In addition, in both datasets, the microRNA-binding regions had similar distribution by mRNA regions (3’UTR, CDS, 5’UTR), and to the distribution of the mRNA parts present in GENCODE (**Figure 1B**). Thus, we conclude that the datasets generated by CLASH and CLEAR-CLIP techniques are comparable.

In the experimentally obtained CLASH and CLEAR-CLIP datasets, we detected 1,153 common miRNA–mRNA interactions, which were built upon combinations of 933 interactions in CLASH and 944 interactions in CLEAR-CLIP. Average length of experimentally obtained interaction was at 37.2 nt +/− 19.4 nt. Eight hundred and sixty-seven interactions which were common for both datasets had the length of overlap of more than 20 nt, with an average length of 45.8 nt +/− 13.9 nt. To evaluate if this overlap reflects biological phenomenon rather than statistical fluke, we performed computational simulation of CLASH and CLEAR-CLIP interactions in transcripts expressed in HEK293 (N = 7,299) and Huh7.5 (N = 4,977), respectively. For these cell lines, a common set of expressed mRNAs (n = 3,044) was reduced to a set of randomly selected nucleotide fragments with the size distribution matching that for nucleotide fragments of CLASH and CLEAR-CLIP; then, we analyzed these sets of sequences for overlap. After five independent runs with randomly selected fragments of matching size distribution, we detected, on average, 7.4 +/− 1.3 interactions with an average length of overlapped segments at 14 nt +/− 6.7 nt. Among these interactions, only a fraction had the length of overlap of more than 20 nt (5.0 +/− 2.5). Therefore, the characteristics of experimentally detected patterns of miRNA–mRNA interactions differ from that of interactions generated by simulation of random events (P < 0.0001).

To investigate whether the low degree of the overlap between miRNA–mRNA interactions registered in CLASH and CLEAR-CLIP datasets could be due to low degree of the overlap between HEK293 and Huh7.5 transcriptomes, expression data collected from these two cell lines were downloaded from GEO repository and analyzed. While about half of expressed microRNAs were found in both these cell lines, an overall difference of HEK293 and Huh7.5 specific sets of highly expressed genes was evident (**Supplementary Figures 1A, B**). To find out if cell-specific differences in microRNA–mRNA interactomes are due to cell-specific environment, the relationships between the levels of expression for individual miRNAs and their targets as well as the patterns of interactions for each mRNA and miRNA in the both cell lines were investigated in details.

### Expression Analysis of microRNA–mRNA Interactome

#### mRNA Expression Analysis

To investigate the degree to which cell-specific levels of transcripts depend on respective microRNAs, we compared expression levels of each gene in HEK293 and Huh7.5 cell lines then cross compared them to sets of experimentally detected microRNA interactions. HEK293 and Huh7.5 cell lines express a total of 15,8k and 14,5k genes, respectively. In each of these two cell lines, approximately 6.9k genes interacted with one or more microRNAs (**Supplementary Figure 1C**). Our analysis highlighted 1–2% of mRNAs with confirmed interactions and no expression detected in respective cell line. We found that only few of these mRNAs had more than 10 interactions each. A majority of them were found to have highly conservative paralogs, which may erroneously align with miRNAs or mRNAs and affect the results of miRNA mapping. A majority of non-expressed mRNAs (about 70%) had only one interaction. It is possible that these mRNAs have been detected as chimeric reads resulting from their protection by AGO protein from ribonucleases. Below, we will describe a few microRNAs that were detected only as a part of chimeras.

**Figure 2:**
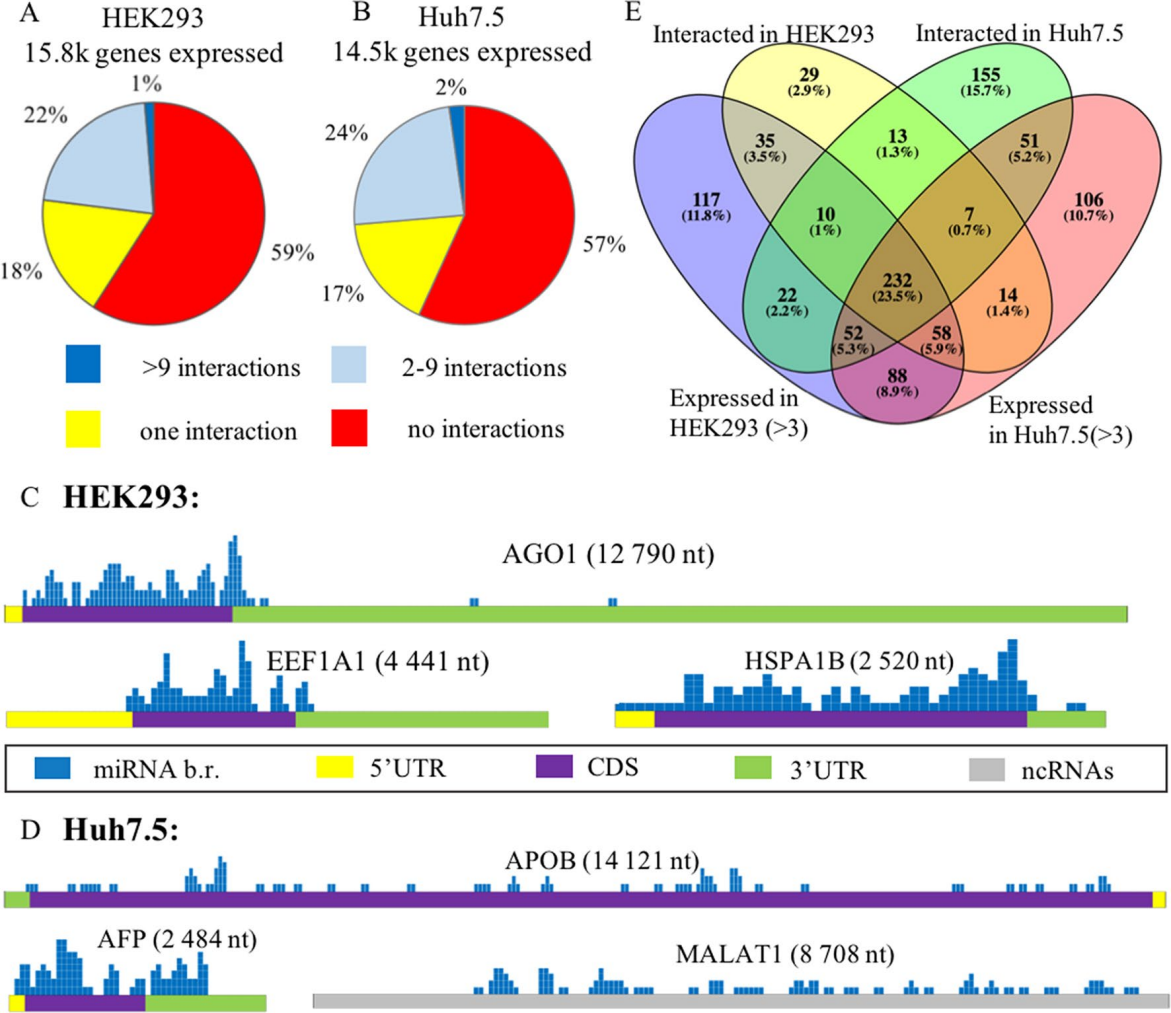
Expression analysis of microRNA and mRNA in HEK293 and Huh7.5 cell lines. **(A)** and **(B)**: Analysis of expressed genes according to amounts of their interactions with microRNAs in HEK293 **(A)** and Huh7.5 **(B)** cell lines. **(C)** and **(D)**: Overview of the microRNA-binding regions locations in sponge-like RNAs expressed in HEK293/CLASH **(C)** and Huh7.5/CLEAR-CLIP **(D)** datasets. After segmenting each of the presented RNAs into 50-nt pieces, the segments that interacted with microRNAs were marked blue on the mRNA map. The height represents the number of interactions detected in each of the segment. For each of the sponge-like RNAs, both name and length are placed above the gene schematics. Colored parts of RNAs are labeled as follows: 5’UTR—yellow, coding region—violet, 3’UTR—green, noncoding region—gray. **(E)** The overlaps between expressed and interacting microRNAs in HEK293 and Huh7.5 cell lines.

In each of these cell lines, a majority of expressed mRNAs (57–59%) did not interact with any microRNA (**Figures 2A, B**). In CLASH and CLEAR-CLIP datasets, there were 215 and 333 high-interacting mRNAs, respectively, with nine or more miRNA interactions for each.

Cell line–specific pie charts built for the miRNA–mRNA interactions per each mRNA were similar. Nevertheless, comparison of the most regulated sets of genes with nine or more interactions each revealed that these sets were cell-line-specific, with only 18 genes in common. These common 18 genes formed in average of 15.7+/−3.2 and 14.1+/− 2.4 interactions with microRNAs in the HEK293 and Huh7.5 cell lines, respectively. Surprisingly, cell line–specific sets of microRNA regulators for each of these genes were completely different. By PANTHER analysis of the common set of genes, we detected enrichment in only one Gene Ontology (GO) category—a molecular function of RNA binding (**Supplementary Table 5**).

Further, we identified a set of mRNAs capable of interaction with many different types of microRNA molecules, with no preference to a particular miRNA. Such behavior of ambiguous interactions with many microRNAs is similar to that of circular RNAs and lncRNAs with “sponge” properties. Among “sponge-like” mRNAs with 50 or more interactions detected in HEK293/CLASH were those encoded by *AGO1*, *EEF1A1*, and *HSPA1B* genes. Peculiarly, in Huh7.5/CLEAR-CLIP, same property has been attributed to different set of mRNA, namely, *APOB*, *AFP*, *MALAT1*, and *XIST*. In mRNAs with “sponge-like” property, microRNA interaction sites were located predominantly in the protein-coding part of the transcript (**Figures 2C, D**).

Remarkably, in HEK293 cells, the most interacting mRNA was the one for AGO1 protein, which had been overexpressed on purpose, as part of CLASH protocol. In this experiment, AGO1-encoding mRNA yielded 88 interactions with a total of 50 different microRNAs. Mean expression levels for AGO1-binding miRNAs were similar to that for all other miRNAs, at 7,279.36 counts *vs.* 7,183.92 counts, respectively. In addition to *AGO1* mRNA, HEK293 cell line expressed two other mRNAs displaying non-specific “sponge-like” effect, *HSPA1B* with 77 interactions to 41 different microRNAs and *EEF1A1* with 50 interactions to 42 microRNAs. Similar to artificially over-expressed AGO1 mRNA, *EEF1A1* also highly expressed in HEK293 cell line (>19K tpm), while another “sponge-like” mRNA *HSPA1B* had expression level equals to 775 tpm.

The set of “sponge-like” mRNAs expressed in Huh7.5 cell line was entirely different. The set of “sponge-like” mRNAs expressed in Huh7.5 cell line was entirely different. We revealed two protein-coding “sponge-like” mRNAS: AFP that formed 47 interactions with 32 different microRNAs, and APOB that also formed 47 interactions with 32 different microRNAs. In set of Huh7.5 “sponge-like” RNAs, two well-described noncoding RNAs were detected: MALAT1 (47 interactions to 27 microRNAs) and XIST (55 interactions to 31 microRNAs). In coherence to expression levels of “sponge-like” mRNAs in HEK293 cell line, we observed difference in expression levels for these mRNAs: *AFP*—more than 19K tpm, *APOB*—358 tpm, *XIST*—202 tpm, and *MALAT1*—80 tpm, while the averages for a gene expressed in Huh7.5 were at 69 tpm.

#### Comparative Analysis of microRNA Expression Levels and Their mRNA-Interacting Properties

To assess the role of microRNAs in the regulation of their target mRNAs, we studied two HEK293 and three Huh7.5 miRNA profiles retrieved from RNAseq datasets deposited in GEO (GSE75136 and GSE74014). For each cell line, only high-quality datasets with very high correlation of miRNA-specific expression levels were selected (Pearson’s correlation r > > 0.99). For each miRNA, we analyzed their cell-line specific levels of expression by R package “DeSeq2” in order to normalize miRNA expression and compared these levels to the sets of experimentally detected microRNA–mRNA interactions retrieved from HEK293/CLASH, and Huh7.5/CLEAR-CLIP datasets microRNA was considered as expressed if it had expression levels of more than three counts (see Methods). Less than a quarter (23.5%) of 989 detected miRNAs was present in both cell lines (**Figure 2E**, **Supplementary Table 6**). Notably, many microRNAs expressed in the HEK293 (N = 205) and Huh7.5 (N = 194) cell lines then failed experimental detection as mRNA-interacting molecules in CLASH or CLEAR-CLIP, respectively.

On the other hand, both CLASH and CLEAR-CLIP datasets included 4–17% of mRNA-interacting microRNAs not detected in respective RNAseq datasets at all. On average, these microRNAs had relatively small amounts of interactions: 2.2+/−0.6 interacting partners for 197 microRNAs present in CLASH dataset but absent in HEK293-based RNAseq and 5.1+/−2.2 interacting partners for 168 miRNAs present in CLEAR-CLIP dataset but absent in Huh7.5-based RNAseq. For comparison, mean amounts of detected interactions across all microRNAs were at 55.8 +/−12.7 for 398 miRNAs of HEK293/CLASH and at 143.5 +/− 28.5 for 542 miRNAs in Huh7.5/CLEAR-CLIP. We could expect that these miRNAs could possibly have a low expression level and, therefore, had not reached a detection cut-off in RNAseq. Alternatively, these miRNAs may be somehow protected from degradation by RISC.

Next, for each cell line, we kept only expressed and interacted microRNAs and evaluated their cell-specific expression level and the amount of interactions in this cell line (**Supplementary Figure 2**). For each cell line, Spearman correlation levels were quite low, at 0.18 and 0.29 in HEK293 (N = 335) and Huh7.5 (N = 342), respectively. For each miRNA, we calculated the cell line–specific ratios (R) of its expression level to amount of detected interactions (**Supplementary Table 6**).

Detailed analysis of this data allowed us to highlight two interesting types of miRNA. Type 1 comprised microRNAs with high expression level and relatively small amount of interactions with respective mRNAs. When the cut-offs for both R and expression levels were set as ranking at 90th percentile or higher, only 16 miRNAs for HEK293 (expression > 4,418 and ratio > 252) and 12 miRNAs in Huh7.5 (expression > 6,941 and ratio > 209) were classified as type 1. Notably, eight type 1 miRNAs were present in both cell lines examined.

Type 2 microRNAs were characterized by a low R, and many detected interactions with mRNAs. When the cut-off for R was set as ranking at 10th percentile or lower, and amounts of interactions at 90^th^ percentile or higher, only 11 and 6 miRNAs for HEK293 (amount of interactions > 150 and ratio < 0.9) and Huh7.5 (amount of interactions > 165 and ratio < 2.5), respectively, were classified as type 2. Unlike the type 1 microRNAs, type 2–specific sets from HEK293 and Huh7.5 did not overlap.

In order to evaluate whether these types of microRNAs are evolutionarily constrained, for all mature microRNAs from miRBase, we calculated the mean of the phyloP conservative values in 20 vertebrates. The average cell line–specific phyloP scores for the type 1 and type 2 microRNAs were similar, at 0.99 and 0.95, respectively. Notably, these scores were higher than the average score value calculated for all known microRNAs (0.24), and the score values for all microRNAs that were identified as expressed or interacted in HEK293 or Huh7.5 cell lines (0.74 and 0.71, respectively). Notably, 80% of top 100 miRBase microRNAs with the highest conservative phyloP scores were seen either as expressed or interacted (or both) in at least one of these two cell lines. On average, in HEK293 and Huh7.5 cells, these most conservative microRNAs had two times higher expression levels than less conservative expressed microRNAs (**Supplementary Table 6**). Overall, higher than average conservativeness of type 1 and type 2 microRNAs may point at the relative importance of their functions.

#### Comparing Cellular Contexts for microRNA’s Interactions

As expected, a majority of microRNAs were concordant in two cell lines: their expression levels and amounts of mRNA interactions were similar in both cellular contexts (**Supplementary Figure 3A**). Nevertheless, some miRNAs have demonstrated remarkable cell specificity in their ratios R (**Supplementary Figures 3B, C**).

For 30 microRNAs, we detected high concordance between their expression level and amount of experimentally detected interactions. Eighteen of these miRNAs had higher expression and mRNA-binding activity in Huh7.5 cell line, while for 12 remaining microRNA, both mRNA-binding activity and expression level were higher in HEK293 cells (**Supplementary Figure 3B**). As an example, in Huh7.5 cell line, expression levels of MAPK1-repressing hsa-miR-194-5p (Kong et al., 2018) were 89 times higher than that in HEK293 cells; in Huh7.5 cells, this microRNA displayed 336 interactions, while in HEK293, it formed only 7 interactions. On the other hand, in HEK293, expression levels of lanosterol synthase suppressing miRNA hsa-miR-10a-5p (Kim et al., 2018) were 450 times higher than that in Huh7.5 cells. In HEK293 cells, this microRNA displayed 267 interactions, while in Huh7.5, it formed only 8 interactions. Such observations were expectable: microRNAs with higher expression level may be capable of the binding to a larger repertoire of targets.

Peculiarly, a total of four microRNAs have performed in exactly opposite way: in cells with higher expression levels, these microRNAs displayed lesser amounts of interactions with their mRNA targets (**Supplementary Figure 3C**). For example, in Huh7.5 cell line, expression levels of hsa-miR-331-3p and hsa-miR-100-5p were at 1,030 and 916 counts, respectively, while in HEK293, these miRNAs had 65 and 41 expression counts, respectively. However, in both cases, amounts of interactions in Huh7.5 cell line were lesser than that in HEK293 cell line, 47 *versus* 342 partners for hsa-miR-331-3p, and 1 *versus* 30 partners for hsa-miR-100-5p. To investigate if this phenomenon is due to the difference in the cell-specific expression levels of target genes, we performed an analysis of all these targets. This was not the case as well. As an example, only 21 out of 318 individual miRNA targets of hsa-miR-331-3p were active in HEK293 cell line but not detected in Huh7.5.

### Analysis of Expanded Set of Experimentally Confirmed microRNA-Binding Regions

Experimentally identified microRNA-binding regions form a promising basis for further queries into the basics of the gene expression regulation and lead to uncovering novel disease-causing mechanisms. To enhance a set of microRNA–mRNA interactions retrieved from CLASH and CLEAR-CLIP studies, we performed the database integration of the data collected in cross-linking with immunoprecipitation (CLIP) experiments that provide information about microRNA-binding regions of target genes but unable to identify mRNA–microRNA pairings.

For this purpose, we collected data from 79 CLIP experiments, comprising 61 HITS-CLIP and 18 PAR-CLIP datasets covering 9 different cell lines, with a majority of these data obtained either in HEK293 (N = 34 datasets) or Huh7.5 (N = 19 datasets) (**Supplementary Table 1**). After combining CLIP datasets with the data of previously mentioned CLASH and CLEAR-CLIP studies, approximately 156,000 unique microRNA-binding regions were catalogued within their respective mRNA targets.

**Figure 3:**
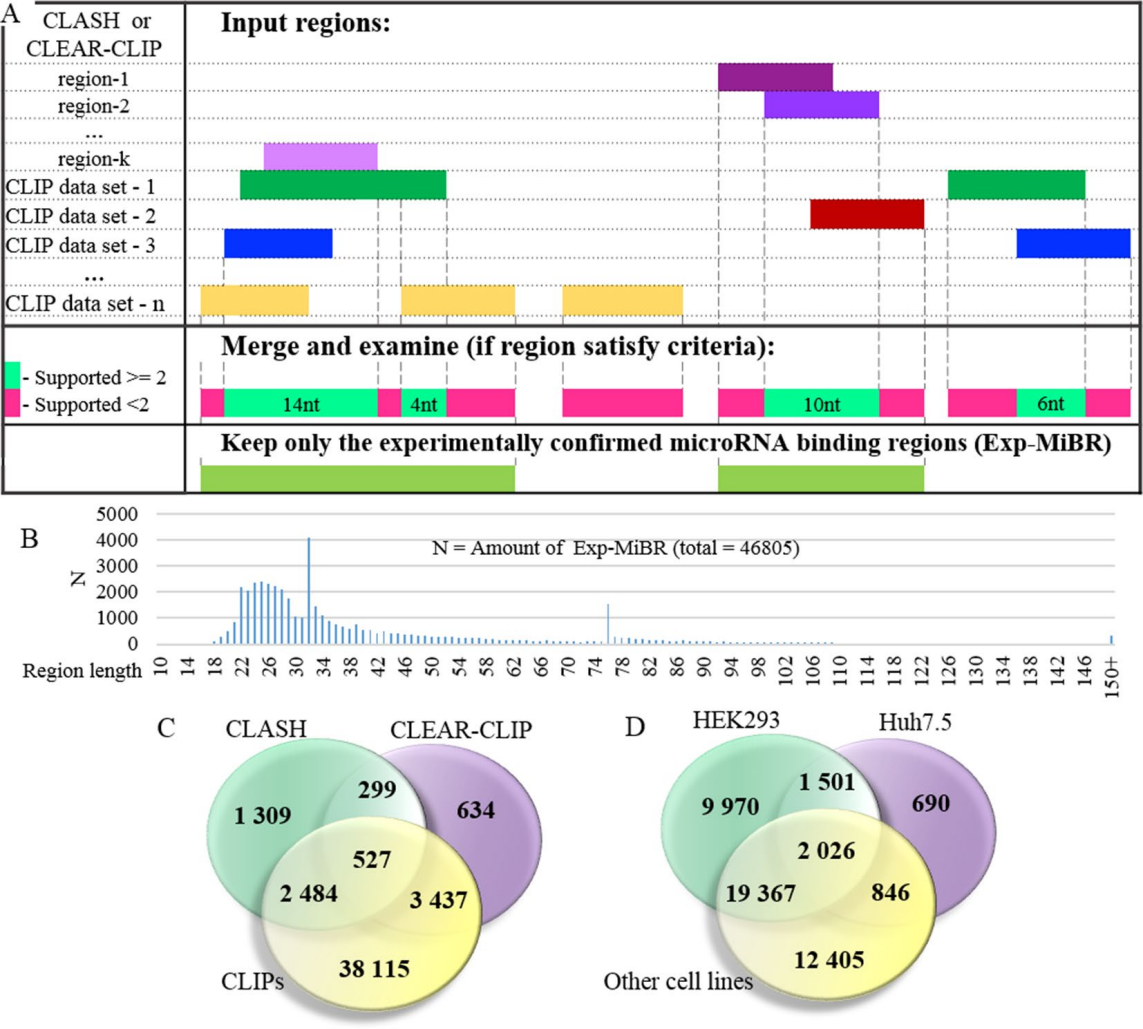
Detailed analysis of experimentally confirmed microRNA-binding regions (Exp-MiBRs). **(A)** Validation of the Exp-MiBR by their independent occurrence in two or more datasets, or in two or more chimeric sequences from one dataset. **(B)** Exp-MiBRs: distribution of the lengths. On horizontal axis—the length of the Exp-MiBRs subsequence; on vertical axis—amounts of the detected Exp-MiBRs (N). **(C)**. Venn diagram depicting Exp-MiBRs detected in experiments employing three different types of identification techniques. **(D)** Venn diagram depicting tissue specificity of Exp-MiBRs detected in HEK293, Huh7.5, and all other cell lines.

At the next stage, the set of microRNA-binding regions was cleaned up to include only these satisfying following criteria: (i) every position in this microRNA-binding subsequence is supported by evidence from at least two different datasets or two different chimeric sequences and (ii) the length of at least 10 nt (**Figure 3A**, **Supplementary Table 3**). MiRNA-binding subsequences of this kind (N = 46,805) formed a dataset of experimentally confirmed microRNA-binding regions (Exp-MiBR). In this dataset, each Exp-MiBR record includes following attributes: genomic coordinates, gene name, type of mRNA part, and list of GEO GSM IDs for experiments which support this microRNA interaction, cellular context, and the list of interacting microRNAs (if accessible). The criteria for inclusion of individual microRNA-binding regions in Exp-MiBR database are justified by analysis presented in **Supplementary Table 2**.

Exp-MiBR subsequences (N = 46,805) were mapped to approximately 15,000 human genes. About one-half of Exp-MiBRs (48%) were located in 3’UTRs, 24% in a coding part of the gene, 10% in introns, and 6% in intergenic parts. Remaining 10% of the Exp-MiBRs were mapped to non-coding RNAs, being matched to either exonic or intronic regions of these loci. For 8,000 of Exp-MIBRs, at least 1 bound microRNA was confirmed by either CLASH or CLEAR-CLIP data (**Figure 3C**).

Approximately 68% of Exp-MiBRs were 20–40 nt in size, closely matching the mean length (33 nt) for all miRNA-binding regions extracted from CLIPs, CLASH, and CLEAR-CLIP data (**Figure 3B**). The second peak in size distribution of Exp-MiBRs was at 75 to 80 nt, being predominantly comprised (86%) of miRNA-interacting region extracted from CLEAR-CLIP dataset. While the sizes of 99% of these Exp-MiBRs were smaller than 150 nt, a few Exp-MiBRs were much longer than that, while remaining supported by many experiments. The longest Exp-MiBR of 631 nt was formed by the regions confirmed as microRNA-interacting in 54 different experiments in nine different cell lines. In addition, there were a few Exp-MiBRs located closely to each other. Such clusters of Exp-MiBRs with many interacting microRNAs do not display a tendency to any particular region of mRNA, as they may be present in CDS, 3’UTR, 5’UTR, or intergenic regions. As an example, chromosome 2 contains a cluster of Exp-MiBRs covering an area of approximately 1.5 kb in size, which is located between the loci of RNA5-8SP5 and MIR663B genes. According to CLASH and CLEAR-CLIP studies, this cluster of Exp-MiBRs interacts with 52 different miRNAs (**Supplementary Figure 4**, **Supplementary Table 7**).

### Tissue-Specific and Housekeeping microRNA-Binding Regions

To characterize Exp-MiBRs further, we analyzed their tissue specificity. Most CLIP experiments were performed either in HEK293 (43%) or in Huh7.5 (24%) cells, while the rest of the CLIP data were collected in HeLa, HFF, BC-1, BC-3, EF3D, LCL35, or LCL cells. In HEK293 cells, we found approximately 9,900 unique MiBRs, while analysis of Huh7.5 cells yielded 690 tissue-specific interacting regions (**Figure 3D**). Larger amounts of Exp-MiBRs in HEK293 as compared to that Huh7.5 cells may be explained either by better coverage of HEK293 transcriptome by various CLIPs (**Supplementary Table 1**), or by intrinsic cell-specific features of miRNA interactomes.

Interestingly, some Exp-MiBRs were observed in a majority of studied cells, possibly reflecting a housekeeping function of these interactions. Approximately 1% of all Exp-MiBRs were found in seven or more cell lines. The functional roles of 351 ubiquitous Exp-MiBRs were investigated using Panther software. The GO analysis showed enrichment of genes participating in cellular process of cell cycle (FС 3.17; p-value 1e10−8) and in molecular function of nucleic acid binding (FC 1.75; p-value 5e10−4).

### Mitochondrial Regulation by microRNA

An analysis of Exp-MiBRs revealed that these microRNA interacting sequences cover 86% of the mitochondrial genome, including 35 out of 37 mitochondrial genes. Mitochondrial Exp-MiBRs (N = 37) were found in all nine investigated cell lines, with each Exp-MiBR discovered, on average, in 11 independent experiments. In total, we identified 182 miRNAs that bind to various mitochondrial RNAs, with two mitochondrial regions binding 107 out of 182 miRNAs.

## Discussion

Experimental identification of microRNA-binding regions is an important prerequisite for querying into the basics of the gene expression regulation, and for uncovering novel disease-causing mechanisms. To date, only two sequencing-based experimental datasets describing full miRNA–mRNA interactomes of human cells, CLASH and CLEAR-CLIP, are available. In both studies, the primary goal was to develop and optimize the experimental protocol itself, while identifying miRNA–mRNA interactions in a particular cell line grown under different conditions. Although these techniques provide a unique window into miRNA targeting, they are not free of limitations, which preclude thorough mapping of entire miRNA–mRNA interactome. Nevertheless, intersecting CLASH and CLEAR-CLIP datasets allowed us to detect much larger set of validated interactions than the overlap of two randomly generated datasets in all five replications. Surprisingly, in both CLASH and CLEAR-CLIP datasets, the distributions of miRNA-binding regions were similar to that in GENCODE transcripts, and more or less even across all types of mRNA regions (3’UTR, CDS or 5’UTR), with no enrichment in miRNA-binding sites within 3’UTRs. Thus, our analysis supports observations of Ragan et al. (Ragan et al., 2009), rather than the model of Grimson et al. (Grimson et al., 2007).

Typically, miRNA–mRNA interaction networks are built *in silico*, with an aid of one or another miRNA prediction tool, and include thousands of mRNA targets. In our study, we attempted painting a holistic picture of human miRNA–mRNA interactome by comparing the entries from experimentally collected datasets describing miRNA-binding activity to the gene expression data. Interestingly, we found that more than half of mRNA transcripts do not bind to any miRNAs present in the same cellular environment. On the other hand, from 1 to 2% of human transcripts interact with nine or more miRNAs, thus, displaying sponge-like activity (Thomson and Dinger, 2016). It was surprising to find that more than half of mRNA transcripts do not bind to any miRNAs present in the same cellular environment. On the other hand, we observed that from 1 to 2% of human transcripts interact with nine or more miRNAs each, thus, displaying sponge-like activity (Thomson and Dinger, 2016). These observations suggest that one can figure out whether some mRNAs may possess such property by analyzing the number of its interactions and the level of its expression: some genes are expressed at a high level but have much fewer interactions than other expressed at same tpm range. This means that each mRNA differs in their miRNA-binding capacities, and some of them do it in more efficient manner than others. Remarkably, observed miRNA–mRNA sponge-like interactions were cell-line-specific, with very little overlap identified. In HEK293 cells, the most prominent sponge-like mRNA, with 77 different miRNA interactions detected, was one encoding for AGO1. In settings of this particular experiment, this mRNA had been overexpressed artificially, as part of CLASH protocol. Two other HEK293-specific “sponge-like” mRNAs, HSPA1B and EEF1A1, formed 77 and 50 interactions, respectively.

For each of these mRNAs, amounts of detected interactions were comparable to that of a well-known circular RNA with sponge properties, Cdr1as (74 predicted sites) (Xu et al., 2015). In Huh7.5 cells, the set of RNAs with “sponge-like” activities included many well-described noncoding RNAs—for example, MALAT1 and XIST. It is peculiar that some Huh7.5-specific sponge-like RNAs, including those for alpha-fetoprotein (AFP) (Parpart et al., 2014) and APOB (Bi et al., 2014), were previously described as biomarkers of liver carcinoma, a tissue of origin for Huh7.5 cell line. In any case, presented set of experimentally identified miRNA–mRNA interactions allows finding a set of endogenous RNAs competing for any particular miRNA.

Some miRNAs expressed at relatively high levels were not among RNA interactors at all. About a hundred of such non-interacting miRNAs were present in both studied cell lines. There is a possibility that the natural targets for these microRNAs are either not expressed in studied cellular contexts, or that they have no targets at all. In total, only 232 microRNAs had at least one interaction in each of studied cell lines.

For individual miRNAs, levels of their expression have no bearing on amounts of interactions they display, possibly reflecting difference in their functions depending on the cellular context. As an example, we revealed that, in Huh7.5 cell line, miR-423-3p is abundant but displays only a few interactions, while in HEK293 cell line, the same miRNA forms more than 200 interactions and expressed at the quite low level. These observations complement previous findings of Mullokandov and colleagues (Mullokandov et al., 2012), who have shown that the binding activity of some highly expressed miRNAs may be weakened by either high target-to-miRNA ratio or the relocation of this miRNA to the nucleus. Further studies are required in order to investigate how RNA binding properties of individual miRNAs may change in response to context-dependent regulation by extrinsic or intrinsic factors.

Augmenting CLASH and CLEAR-CLIP datasets with additional 79 CLIP datasets provided us with information about microRNA footprints of many thousands of experimentally confirmed microRNA-binding regions (Exp-MiBRs) distributed through both coding and noncoding regions of RNA loci. At least some Exp-MiBRs are tissue-specific, in agreement with Clark and colleagues, who revealed the differences in the microRNA targetomes across tissues (Clark et al., 2014).

In addition to chromosomes, many Exp-MiBRs map to mitochondrial DNA, where they are quite abundant. Previous studies showed four mitochondrial regions with high degree of homology to microRNAs, namely, hsa-miR-4461 (chrM: 10,690–10,712), hsa-miR-4463 (chrM: 13,050–13,068), hsa-miR-4484 (chrM: 5,749–5,766), and hsa-miR-4485 (chrM: 2,562–2,582) (Sripada et al., 2012). Two of these regions encode mitochondrial *ND4L* and 16S rRNA genes and correspond to highly interacting Exp-MiBRs, with 70 and 63 cognate miRNAs, respectively, all confirmed in nine different cell lines. In both cases, previously identified cognate miRNAs hsa-miR-4461 and hsa-miR-4485 were among confirmed interactors. Our study expands the coverage of mitochondrial genome by various miRNA-interacting regions to 86% of its lengths. Altogether, these findings support the notion that miRNA–mRNA interactions take place in a variety of cellular compartments, including mitochondria (Ni and Leng, 2015).

The landscape of microRNA–mRNA human interactions, which we derived from both direct microRNA–mRNA interactions experimentally defined in HEK293 and Huh7.5 cell lines, when analyzed along with microRNA and mRNA expression data, highlights enormous complexity of human microRNA–mRNA interactome. For individual miRNAs, levels of their expression have no bearing on amounts of interactions they display, possibly reflecting context depending difference in their functions. In this article, we show that, while only 1–2% of human genes are highly regulated by microRNAs, a few cell-specific RNAs display sponge-like effects, including *EEF1A1* and *HSPA1B* in HEK293 and *AFP*, *APOB*, and *MALAT1* genes in Huh7.5 cell lines. Some miRNAs might be expressed at relatively low levels and interact with many mRNAs. On the other hand, there is a set of microRNAs expressed at a very high level and interacting with only a few mRNAs, thus, indeed, regulating expression of their targets in a specific manner. Notably, microRNAs are capable of switching between these two modes of action, depending on cellular context. The question of the biological significance of these two miRNA groups remains open. CLASH and/or CLEAR-CLIP coverage of additional cell lines is warranted. It is notable, however, that the presence of miRNA groups, one with a low expression level and a high number of interactions, and one with opposite characteristics, was independently detected in both cell lines profiled.

We have also established a collection of reliable microRNA-binding regions that we systematically extracted in course of an analysis of 79 CLIP datasets. This collection is available at http://score.generesearch.ru/services/mirna/. The promise of microRNAs as potential diagnostic mean and therapeutic target got expanded with a number of pathogenic loss-of-function and, recently, gain-of-function mutations described (Grigelioniene et al., 2019). Hence, our efforts in mapping the human miRNA–mRNA interactome may be aided in untangling molecular underpinnings of hereditary and acquired diseases.

## Data Availability Statement

microRNA–mRNA interactome data were extracted from published CLASH (Helwak et al., 2013) and CLEAR-CLIP (Moore et al., 2015) studies. Publicly available datasets of RNA and microRNA expression were from GEO accessions “GSE68611” (Murakawa et al., 2015), “GSE64677” (Luna et al., 2015), “GSE75136” (Wissink et al., 2016), “GSE74014” (Bandiera et al., 2016). GEO IDs’ of open-accessed Raw CLIP datasets are listed as **Supplementary Table 3**. All data generated during this study are included in this published article and its supplementary information files.

## Author Contributions

MS and OP designed the study and carried out the research. AB contributed to the discussion of the results. OP and AB wrote the paper. All authors read and approved the final manuscript.

## Funding

This project has been funded in part by the Laboratory of functional genomics of the Research Centre for Medical Genetics and by the Laboratory of functional genome analysis of the Moscow Institute of Physics and Technology.

## Conflict of Interest

The authors declare that the research was conducted in the absence of any commercial or financial relationships that could be construed as a potential conflict of interest.

## Abbreviations

AGO, Argonaute; CDS, coding DNA sequence; CLASH, cross-linking, ligation, and sequencing of hybrids technique; CLEAR-CLIP, covalent ligation of endogenous Argonaute-bound RNA-CLIP technique; CLIP, UV cross-linking and immunoprecipitation technique; Exp-MiBRs, experimentally confirmed microRNA-binding regions; HITS-CLIP, high-throughput sequencing of RNA isolated by cross-linking immunoprecipitation; iCLIP, individual-nucleotide resolution cross-linking and immunoprecipitation; PAR-CLIP, photoactivatable-ribonucleoside-enhanced immunoprecipitation; UTR, untranslated region.

## References

[B1] Agarwal V. Bell G. W. Nam J. W. Bartel D. P. (2015). Predicting effective microRNA target sites in mammalian mRNAs. Elife 4, e05005. 10.7554/eLife.05005 PMC4532895 26267216

[B2] Artcibasova A. V. Korzinkin M. B. Sorokin M. I. Shegay P. V. Zhavoronkov A. A. Gaifullin N. (2016). MiRImpact, a new bioinformatic method using complete microRNA expression profiles to assess their overall influence on the activity of intracellular molecular pathways. Cell Cycle 15 (5), 689–698. 10.1080/15384101.2016.1147633 27027999 PMC4845938

[B3] Bandiera S. Pernot S. El Saghire H. Durand S. C. Thumann C. Crouchet E. (2016). Hepatitis C virus-induced upregulation of microRNA miR-146a-5p in hepatocytes promotes viral infection and deregulates metabolic pathways associated with liver disease pathogenesis. J. Virol. 90 (14), 6387–6400. 10.1128/JVI.00619-16 27147737 PMC4936128

[B4] Bartel D. P. (2004). MicroRNAs: genomics, biogenesis, mechanism, and function. Cell 116 (2), 281–297. 10.1016/S0092-8674(04)00045-5 14744438

[B5] Bi Y. He Y. Huang J. Su Y. Zhu G. H. Wang Y. (2014). Functional characteristics of reversibly immortalized hepatic progenitor cells derived from mouse embryonic liver. Cell. Physiol. Biochem. 34 (4), 1318–1338. 10.1159/000366340 25301359

[B6] Bray N. L. Pimentel H. Melsted P. Pachter L. (2016). Near-optimal probabilistic RNA-seq quantification. Nat. Biotechnol. 34 (5), 525–527. 10.1038/nbt.3519 27043002

[B7] Clark P. M. Loher P. Quann K. Brody J. Londin E. R. Rigoutsos I. (2014). Argonaute CLIP-Seq reveals miRNA targetome diversity across tissue types. Sci. Rep. 4, 5947. 10.1038/srep05947 25103560 PMC4894423

[B8] Corcoran D. L. Georgiev S. Mukherjee N. Gottwein E. Skalsky R. L. Keene J. D. (2011). PARalyzer: definition of RNA binding sites from PAR-CLIP short-read sequence data. Genome Biol. 12 (8), R79. 10.1186/gb-2011-12-8-r79 21851591 PMC3302668

[B9] Frankish A. Diekhans M. Ferreira A. M. Johnson R. Jungreis I. Loveland J. (2018). GENCODE reference annotation for the human and mouse genomes. Nucleic Acids Res. 47 (D1), D766–D773. 10.1093/nar/gky955 PMC6323946 30357393

[B10] Friedman R. C. Farh K. K. H. Burge C. B. Bartel D. P. (2009). Most mammalian mRNAs are conserved targets of microRNAs. Genome Res. 19 (1), 92–105. 10.1101/gr.082701.108 18955434 PMC2612969

[B11] Grigelioniene G. Suzuki H. I. Taylan F. Mirzamohammadi F. Borochowitz Z. U. Ayturk U. M. (2019). Gain-of-function mutation of microRNA-140 in human skeletal dysplasia. Nat. Med. 1, 583. 10.1038/s41591-019-0353-2 PMC6622181 30804514

[B12] Grimson A. Farh K. K. H. Johnston W. K. Garrett-Engele P. Lim L. P. Bartel D. P. (2007). MicroRNA targeting specificity in mammals: determinants beyond seed pairing. Mol. Cell. 27 (1), 91–105. 10.1016/j.molcel.2007.06.017 17612493 PMC3800283

[B13] Gumienny R. Zavolan M. (2015). Accurate transcriptome-wide prediction of microRNA targets and small interfering RNA off-targets with MIRZA-G. Nucleic Acids Res. 43 (3), 1380–1391. 10.1093/nar/gkv050 25628353 PMC4330396

[B14] He L. Hannon G. J. (2004). MicroRNAs: small RNAs with a big role in gene regulation. Nat. Rev. Genet. 5 (7), 522–531. 10.1038/nrg1379 15211354

[B15] Heberle H. Meirelles G. V. da Silva F. R. Telles G. P. Minghim R. (2015). InteractiVenn: a web-based tool for the analysis of sets through Venn diagrams. BMC Bioinformatics 16, 169. 10.1186/s12859-015-0611-3 25994840 PMC4455604

[B16] Helwak A. Kudla G. Dudnakova T. Tollervey D. (2013). Mapping the human miRNA interactome by CLASH reveals frequent noncanonical binding. Cell 153 (3), 654–665. 10.1016/j.cell.2013.03.043 23622248 PMC3650559

[B17] Hu B. Yang Y. C. T. Huang Y. Zhu Y. Lu Z. J. (2016). POSTAR: a platform for exploring post-transcriptional regulation coordinated by RNA-binding proteins. Nucleic Acids Res. 45 (D1), D104–D114. 10.1093/nar/gkw888 28053162 PMC5210617

[B18] Jonas S. Izaurralde E. (2015). Towards a molecular understanding of microRNA-mediated gene silencing. Nat. Rev. Genet. 16 (7), 421–433. 10.1038/nrg3965 26077373

[B19] Karolchik D. Hinrichs A. S. Furey T. S. Roskin K. M. Sugnet C. W. Haussler D. (2004). The UCSC Table Browser data retrieval tool. Nucleic Acids Res. 32 (Database issue), D493–D496. 10.1093/nar/gkh103 14681465 PMC308837

[B20] Kim J. E. Hong J. W. Lee H. S. Kim W. Lim J. Cho Y. S. (2018). Hsa-miR-10a-5p downregulation in mutant UQCRB-expressing cells promotes the cholesterol biosynthesis pathway. Sci. Rep. 8 (1), 12407. 10.1038/s41598-018-30530-6 30120311 PMC6098055

[B21] Kong Q. Zhang S. Liang C. Zhang Y. Kong Q. Chen S. (2018). LncRNA XIST functions as a molecular sponge of miR-194-5p to regulate MAPK1 expression in hepatocellular carcinoma cell. J. Cell. Biochem. 119 (6), 4458–4468. 10.1002/jcb.26540 29227532

[B22] Kozomara A. Griffiths-Jones S. (2014). miRBase: annotating high confidence microRNAs using deep sequencing data. Nucleic Acids Res. 42 (Database issue), D68–D73. 10.1093/nar/gkt1181 24275495 PMC3965103

[B23] Kuhn R. M. Haussler D. Kent W. J. (2012). The UCSC genome browser and associated tools. Brief. Bioinformatics 14 (2), 144–161. 10.1093/bib/bbs038 22908213 PMC3603215

[B24] Li Y. Zhang Z. (2014). Potential microRNA-mediated oncogenic intercellular communication revealed by pan-cancer analysis. Sci. Rep. 4, 7097. 10.1038/srep07097 25403569 PMC4235308

[B25] Li Y. Liang C. Wong K. C. Jin K. Zhang Z. (2014). Inferring probabilistic miRNA–mRNA interaction signatures in cancers: a role-switch approach. Nucleic Acids Res. 42 (9), e76–e76. 10.1093/nar/gku182 24609385 PMC4027195

[B26] Licatalosi D. D. Mele A. Fak J. J. Ule J. Kayikci M. Chi S. W. (2008). HITS-CLIP yields genome-wide insights into brain alternative RNA processing. Nature 456 (7221), 464–469. 10.1038/nature07488 18978773 PMC2597294

[B27] Love M. I. Huber W. Anders S. (2014). Moderated estimation of fold change and dispersion for RNA-seq data with DESeq2. Genome Biol. 15 (12), 550. 10.1186/s13059-014-0550-8 25516281 PMC4302049

[B28] Lu Y. Leslie C. S. (2016). Learning to predict miRNA-mRNA interactions from AGO CLIP sequencing and CLASH data. PLoS Comput. Biol. 12 (7), e1005026. 10.1371/journal.pcbi.1005026 27438777 PMC4954643

[B29] Luna J. M. Scheel T. K. Danino T. Shaw K. S. Mele A. Fak J. J. (2015). Hepatitis C virus RNA functionally sequesters miR-122. Cell 160 (6), 1099–1110. 10.1016/j.cell.2015.02.025 25768906 PMC4386883

[B30] Moore M. J. Scheel T. K. Luna J. M. Park C. Y. Fak J. J. Nishiuchi E. (2015). miRNA-target chimeras reveal miRNA 3’-end pairing as a major determinant of argonaute target specificity. Nat. Commun. 6, 8864. 10.1038/ncomms9864 26602609 PMC4674787

[B31] Moore M. J. Zhang C. Gantman E. C. Mele A. Darnell J. C. Darnell R. B. (2014). Mapping argonaute and conventional RNA-binding protein interactions with RNA at single-nucleotide resolution using HITS-CLIP and CIMS analysis. Nat. Protoc. 9 (2), 263–293. 10.1038/nprot.2014.012 24407355 PMC4156013

[B32] Mullokandov G. Baccarini A. Ruzo A. Jayaprakash A. D. Tung N. Israelow B. (2012). High-throughput assessment of microRNA activity and function using microRNA sensor and decoy libraries. Nat. Methods. 9 (8), 840–846. 10.1038/nmeth.2078 22751203 PMC3518396

[B33] Murakawa Y. Hinz M. Mothes J. Schuetz A. Uhl M. Wyler E. (2015). RC3H1 post-transcriptionally regulates A20 mRNA and modulates the activity of the IKK/NF-κB pathway. Nat. Commun. 6, 7367. 10.1038/ncomms8367 26170170 PMC4510711

[B34] Ni W. J. Leng X. M. (2015). Dynamic miRNA-mRNA paradigms: new faces of miRNAs. Biochem. Biophys. Rep. 4, 337–341. 10.1016/j.bbrep.2015.10.011 29124222 PMC5669400

[B35] Parpart S. Roessler S. Dong F. Rao V. Takai A. Ji J. (2014). Modulation of miR-29 expression by α-fetoprotein is linked to the hepatocellular carcinoma epigenome. Hepatology 60 (3), 872–883. 10.1002/hep.27200 24798303 PMC4146718

[B36] Plotnikova O. M. Skoblov M. Y. (2018). Efficiency of the miRNA-mRNA interaction prediction programs. Mol. Biol. (Mosk.) 52 (3), 543–554. 10.7868/S0026898418030187 29989587

[B37] Ragan C. Cloonan N. Grimmond S. M. Zuker M. Ragan M. A. (2009). Transcriptome-wide prediction of miRNA targets in human and mouse using FASTH. PLoS One 4 (5), e5745. 10.1371/annotation/e0842765-3cae-4737-8b5b-96aeb12d7fb5 19478946 PMC2684643

[B38] Riffo-Campos Á. Riquelme I. Brebi-Mieville P. (2016). Tools for sequence-based miRNA target prediction: what to choose? Int. J. Mol. Sci. 17 (12), 1987. 10.3390/ijms17121987 PMC5187787 27941681

[B39] Sætrom P. Heale B. S. Snøve O. Jr. Aagaard L. Alluin J. Rossi J. J. (2007). Distance constraints between microRNA target sites dictate efficacy and cooperativity. Nucleic Acids Res. 35 (7), 2333–2342. 10.1093/nar/gkm133 17389647 PMC1874663

[B40] Selbach M. Schwanhäusser B. Thierfelder N. Fang Z. Khanin R. Rajewsky N. (2008). Widespread changes in protein synthesis induced by microRNAs. Nature 455 (7209), 58–63. 10.1038/nature07228 18668040

[B41] Sripada L. Tomar D. Prajapati P. Singh R. Singh A. K. Singh R. (2012). Systematic analysis of small RNAs associated with human mitochondria by deep sequencing: detailed analysis of mitochondrial associated miRNA. PLoS One 7 (9), e44873. 10.1371/journal.pone.0044873 22984580 PMC3439422

[B42] Steinkraus B. R. Toegel M. Fulga T. A. (2016). Tiny giants of gene regulation: experimental strategies for microRNA functional studies. Wiley Interdiscip. Rev. Dev. Biol. 5 (3), 311–362. 10.1002/wdev.223 26950183 PMC4949569

[B43] Thomson D. W. Dinger M. E. (2016). Endogenous microRNA sponges: evidence and controversy. Nat. Rev. Genet. 17 (5), 272–283. 10.1038/nrg.2016.20 27040487

[B44] Uhlmann S. Mannsperger H. Zhang J. D. Horvat E. Á. Schmidt C. Küblbeck M. (2012). Global microRNA level regulation of EGFR-driven cell-cycle protein network in breast cancer. Mol. Syst. Biol. 8, 570. 10.1038/msb.2011.100 22333974 PMC3293631

[B45] Wang K. Li M. Hakonarson H. (2010). ANNOVAR: functional annotation of genetic variants from high-throughput sequencing data. Nucleic Acids Res. 38 (16), e164. 10.1093/nar/gkq603 20601685 PMC2938201

[B46] Weiss C. N. Ito K. (2017). A macro view of microRNAs: the discovery of microRNAs and their role in hematopoiesis and hematologic disease. Int. Rev. Cell Mol. Biol. 334, 99–175. 10.1016/bs.ircmb.2017.03.007 28838543 PMC5663456

[B47] Wissink E. M. Fogarty E. A. Grimson A. (2016). High-throughput discovery of post-transcriptional cis-regulatory elements. BMC Genomics 17, 177. 10.1186/s12864-016-2479-7 26941072 PMC4778349

[B48] Xu H. Guo S. Li W. Yu P. (2015). The circular RNA Cdr1as, *via* miR-7 and its targets, regulates insulin transcription and secretion in islet cells. Sci. Rep. 5, 12453. 10.1038/srep12453 26211738 PMC4515639

[B49] Yang H. Wang K. (2015). Genomic variant annotation and prioritization with ANNOVAR and wANNOVAR. Nat. Protoc. 10 (10), 1556–1566. 10.1038/nprot.2015.105 26379229 PMC4718734

[B50] Yates A. Beal K. Keenan S. McLaren W. Pignatelli M. Ritchie G. R. (2014). The Ensembl REST API: Ensembl data for any language. Bioinformatics 31 (1), 143–145. 10.1093/bioinformatics/btu613 25236461 PMC4271150

