# Supplementary material for: Comprehensive Analysis of Human microRNA–mRNA Interactome
Source: Front Genet. 2019 Oct 8;10:933. doi: 10.3389/fgene.2019.00933 (PMC6792129; doi:10.3389/fgene.2019.00933)
Supplement: Supplementary file 8 [file DataSheet_1.pdf]

SUPPLEMENTARY

Supplementary Tables:

Supplementary Table 1. List of 79 CLIP studies utilized.

Supplementary Table 2. Validation of the criteria for selection of experimentally confirmed microRNA binding regions (Exp-MiBRs) (i) *length of subsequence (nt) (ii) every position in all subsequences forming a region is supported by N datasets or chimeras. Exp-MiBR which were utilized for subsequent analysis (N = 46, 8065) are highlighted in green.*

Supplementary Table 3. List of all high confidence microRNA binding regions (total – 46,805) along with the characteristics of each of these regions. Sheets 1: hg 19; Sheet 2: hg38

Supplementary Table 4. Summary of microRNA-mRNA interactome data.

Supplementary Table 5. GO Panther analysis of 18 RNAs characterized by largest amounts of interactions with miRNAs in HEK293 and Huh7.5

Supplementary Table 6. List of miRNAs from CLASH/HEK293 and CLEAR-CLIP/Huh7.5 experiments (N = 989), along with their characteristics: expression levels, amount of interactions, groups, phyloP scores

Supplementary Table 7. Exp-MiBR with known interacted microRNAs form supercluster located between MIR663 and RNA5-8SP5 genes

Supplementary Figures:

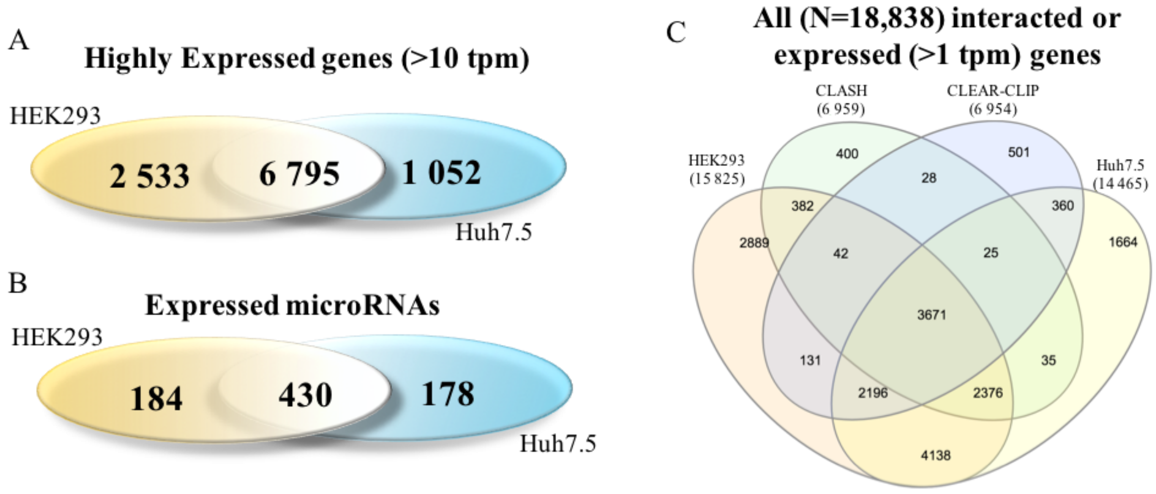

Supplementary Figure 1. Expression patterns of HEK293 and Huh7.5 cell lines. (A) Venn diagram of HEK293- and Huh7.5-specific highly expressed (more than 10 tpm) gene sets covered by CLASH and CLEAR-CLIP interactomes, respectively. **(B)** Venn diagram of HEK293- and Huh7.5-expressed miRNAs represented in CLASH and CLEAR-CLIP interactomes, respectively. **(C)** miRNA interacting RNAs expressed in both HEK293 and Huh7.5 cells according to the intersection of CLASH and CLEAR-CLIP data.

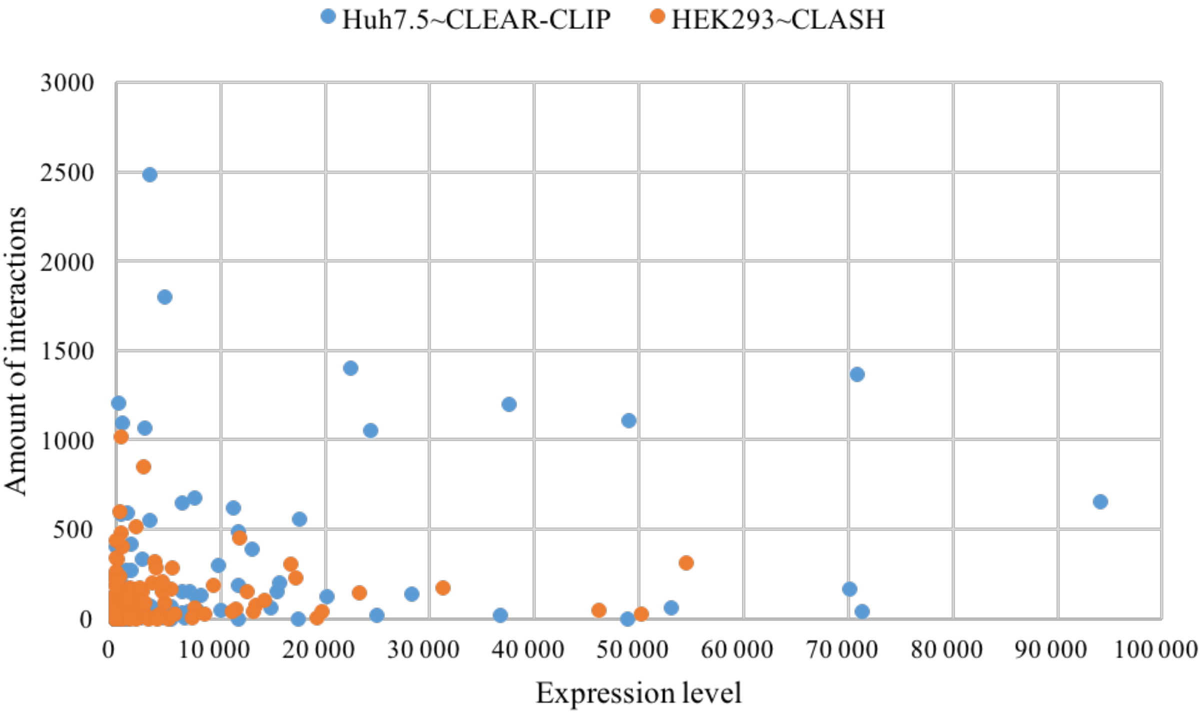

microRNA	Huh7.5 Expression	Interactions Huh7.5	HEK293 Expression	Interactions HEK293
hsa-miR-10a-5p	2478,52	8	1117692,81	267
hsa-miR-10b-5p	140865,59	8	865836,37	108
hsa-miR-191-5p	123800,95	191	113472,34	35
hsa-miR-92a-3p	57830,64	1489	172728,81	1062
hsa-miR-181a-5p	104018,92	337	15209,19	42
hsa-miR-192-5p	190112,21	431	10309,81	3
hsa-miR-22-3p	199116,62	56	9853,84	27

Supplementary Figure 2. Expression levels of studied miRNAs. For each miRNA, the amounts of interactions with mRNA targets plotted against their expression levels. Blue: expression levels in Huh7.5 cells and amounts of interactions identified by CLEAR-CLIP; orange - expression levels in HEK293 cells and amounts of interactions identified by CLASH. Only miRNAs with expression levels of lesser than 100,000 counts in both of the cell lines are presented here. The characteristics of 7 remaining miRNAs are resented in the table below.

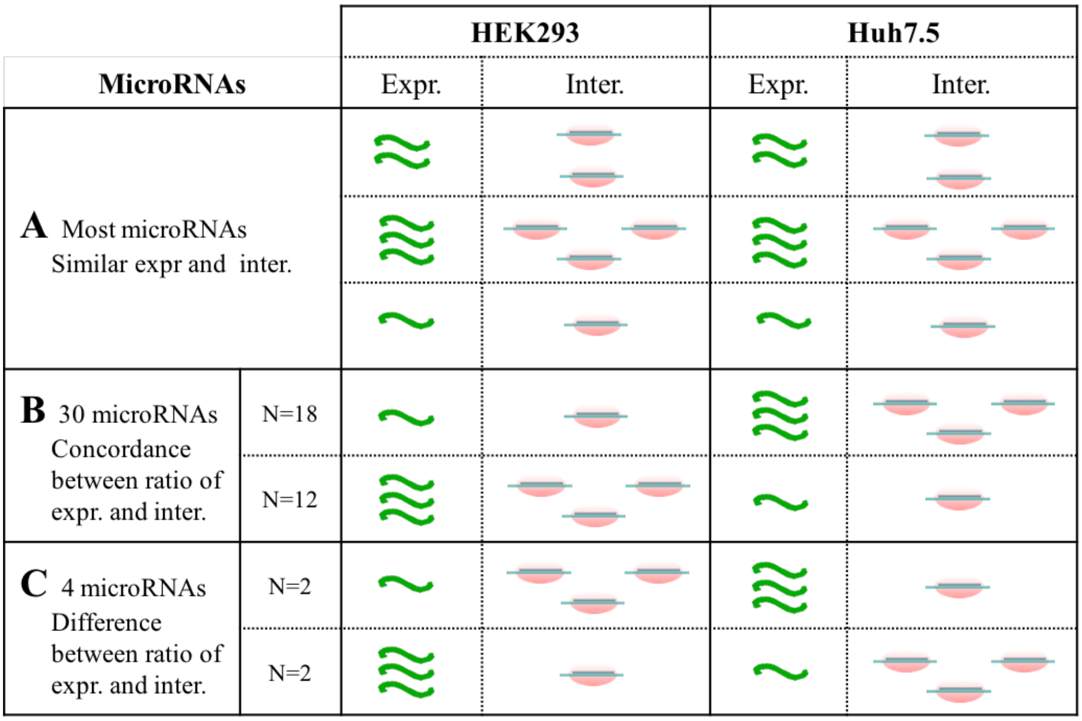

Supplementary Figure 3 RNA-interacting miRNAs may be separated into three groups: miRNAs with similar expression levels and amounts of interactions in both HEK293 and Huh7.5 cell lines (A); miRNAs with greater expression levels and amounts of interactions in one cell line as compared to another (B); miRNAs with substantially larger expression levels in one cell line while interacting with greater amounts of RNAs in another cell line (C). MiRNA expression levels are depicted as a green line(s). The amounts of microRNA-mRNA interactions are shown in the column “Inter.”. Expr. = expression levels; Inter. = amount of interactions.

A

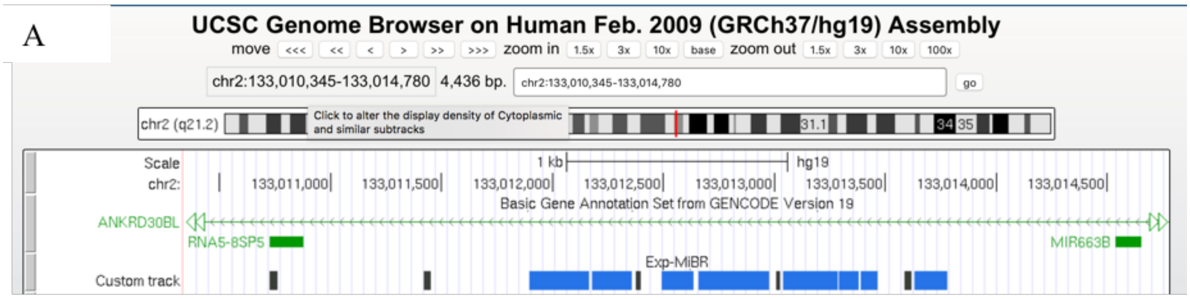

B Number of experimentally confirmed microRNA binding regions

	exonic	3'UTR
AGO1	21	8
AGO2	7	3
AGO3	0	13
AGO4	0	6

Supplementary Figure 4. (A) The cluster of the high confidence microRNA binding regions located between MIR663B and RNA5-8SP5 genes. Exp-MiBRs with experimentally confirmed miRNA interactions shown in blue. Exp-MiBRs supported only by CLIP data shown in grey. (B) Amounts of Exp-MiBRs in loci encoding the proteins of AGO family.
